# Glucagon-like peptide-1 analogues - an efficient therapeutic option for the severe insulin resistance of lipodystrophic syndromes: two case reports

**DOI:** 10.1186/s13256-016-1175-1

**Published:** 2017-01-13

**Authors:** Joana Oliveira, Eva Lau, Davide Carvalho, Paula Freitas

**Affiliations:** 1 Department of Endocrinology, Diabetes and Metabolism, Centro Hospitalar São João, Alameda Prof. Hernâni Monteiro, 4200 Porto, Portugal; 2 Faculty of Medicine, University of Porto, Porto, Portugal

**Keywords:** Lipodystrophy, Diabetes mellitus, GLP-1 analogues

## Abstract

**Background:**

Lipodystrophic syndromes are uncommon medical conditions which are normally associated with metabolic disorders, such as diabetes mellitus, dyslipidemia, and fatty liver disease. These complications are generally difficult to treat, particularly diabetes, due to severe insulin resistance. We present two case reports of successful treatment of diabetes with glucagon-like peptide-1 analogues in patients with clinical features of lipodystrophic syndromes.

**Case presentation:**

Two white women aged 49 and 60 years manifested marked central body fat deposition with severe lipoatrophy of their limbs and buttocks and pronounced acanthosis nigricans. They had hypertension, dyslipidemia, fatty liver disease, and poorly controlled diabetes (glycated hemoglobin 8.3% and 10.2%, respectively) despite the use of three classes of oral antidiabetic drugs taken in combination in the first case, and high doses of insulin in the second case. Four months after the addition of glucagon-like peptide-1 analogue to their previous treatment they both showed a pronounced improvement in metabolic control (glycated hemoglobin 5.6% and 6.2%, respectively). In the first case, a weight loss of nearly 30 kg was recorded.

**Conclusions:**

We intend to demonstrate that glucagon-like peptide-1 analogues could be a valuable tool for patients with lipodystrophic disorders, probably by improving body fat distribution, with favorable results in insulin-sensitivity and glycemic control.

## Background

Lipodystrophic syndromes are rare disorders characterized by selective loss of adipose tissue, mainly from subcutaneous compartments. Lipoatrophy may vary from being partial, co-existing with adipose tissue depots in ectopic sites, to generalized [1]. These syndromes are usually linked with severe metabolic complications, such as insulin resistance, diabetes mellitus, dyslipidemia, hepatic steatosis, and hypertension [2, 3]. Loss of adipose tissue seems to give rise to both lipodystrophy and metabolic disorders [2], which suggests that it is the absence of fat tissue and the consequent leptin deficiency that leads to insulin resistance [4, 5]. Furthermore, adipocytes provide a benign location to accumulate lipids and, thus, when they are absent, lipids will store in the liver, muscle, and ectopic tissues, causing significant metabolic complications [6]. Patients may present acanthosis nigricans, acromegaloid features, muscular hypertrophy, and hirsutism in the context of ovarian hyperandrogenism: polycystic ovary syndrome (PCOS) [2, 7]. Over the last decades, several causative genetic mutations have been identified in patients with lipodystrophies. Nevertheless, lipodystrophy is a clinical diagnosis which is based on a physical examination, and many such patients present no mutations of identified genes, suggesting that other genes are involved and have not yet been deciphered [8]. The treatment of complications involves classical intervention strategies, however, owing to the severity of insulin resistance, managing such complications can be challenging. Glucagon-like peptide-1 (GLP-1) analogues stimulate glucose-dependent insulin secretion and suppress inappropriately elevated glucagon secretion, thus improving glucose homeostasis. These analogues also delay gastric emptying and act centrally to promote satiety, thus reducing food intake, which leads to weight loss. This loss seems to result from a reduction in fat mass, rather than lean tissue mass [9], with a preferential decrease in visceral fat [10]. We describe two clinical cases of diabetes in patients with lipodystrophic features who were successfully treated with GLP-1 analogues.

## Case presentation

### Case 1

A 49-year-old white woman was referred to our Endocrinology department for obesity. She presented diabetes treated with metformin 2500 mg/day, sitagliptin 100 mg/day, and gliclazide 120 mg/day. She had hypertension, dyslipidemia, hyperuricemia, fatty liver disease, and obstructive sleep apnea. Furthermore, she was being treated for a major depression, partly motivated by body image problems. She weighed 89.5 kg, had a body mass index (BMI) of 33.7 kg/m^2^, and presented an evident abdominal fat deposition (waist circumference 123 cm), with markedly reduced adipose tissue in her limbs and buttocks. She had a round face with cervical acanthosis nigricans, and an accumulation of fat in the chin and in the cervicodorsal and supraclavicular regions. Laboratory tests revealed glycated hemoglobin (A1c) 8.3%; total cholesterol 273 mg/dL (<200), high-density lipoprotein (HDL) cholesterol 49 mg/dL (>60), low-density lipoprotein (LDL) cholesterol 196 mg/dL (<130), triglycerides 350 mg/dL (<150), under statin; and uric acid 6.9 mg/dL (2.3 to 6.1). She presented normal 24-hour urinary free cortisol of 39 mcg/24 hours (36 to 137) and an overnight dexamethasone suppression test recorded a serum cortisol of 0.4 mcg/dL (<1.8mcg/dL). She started taking exenatide 2 mg once a week, with good tolerance. Sitagliptin was suspended, while maintaining the other antidiabetic drugs. After 4 months of therapy, she lost 29.2 kg (approximately 33% of her initial body weight) and her A1c was 5.6%. The results of genetic tests for mutations in *LMNA* gene and *PPAR-gamma* gene were negative.

### Case 2

A 60-year-old white woman was observed in our Endocrinology department for diabetes. She presented hypertension, dyslipidemia, fatty liver disease, severe obstructive sleep apnea, depressive disorder, and a history of a previous ischemic stroke. She had poor glycemic control, with A1c values between 9 and 11% over recent years, requiring several medication adjustments. She was treated with metformin 2000 mg/day, and a total daily dose of insulin of nearly 240 units in a basal-bolus regimen. Her regimen was: neutral protamine Hagedorn (NPH) insulin three times daily (60 units in the morning, 50 units at lunch, and 46 units at night); insulin aspart at mealtimes (21 units at breakfast, 24 units at lunch, 11 units at afternoon snack, and 27 units at dinner); and a correctional insulin dose was added, based on her pre-prandial glucose value, resembling a sliding scale. Her insulin sensitivity factor was 10. Her physical examination revealed that she was overweight (weight 75.9 kg, BMI 33.7 kg/m^2^), with central body fat deposition (waist circumference 105 cm) and prominent lipoatrophy of her limbs and buttocks. She presented pronounced cervical and axillary acanthosis nigricans, and hirsutism. Laboratory tests showed an A1c of 10.2% and dyslipidemia: total cholesterol 226 mg/dL (<200), HDL cholesterol 51 mg/dL (>60), LDL cholesterol 131 mg/dL (<130), triglycerides 221 mg/dL (<150), despite being medicated with statin. She had mildly elevated liver enzymes levels: aspartate aminotransferase 67 U/L (10 to 31), alanine aminotransferase 76 U/L (10 to 31), gamma glutamyl transferase 321 U/L (7 to 32), and alkaline phosphatase 175 U/L (30 to 120). Her renal function was normal. Cushing syndrome had been excluded: 24-hour urinary free cortisol 80.1 mcg/24 hours (36 to 137). An overnight dexamethasone suppression test registered serum cortisol of 0.8 mcg/dL (<1.8 mcg/dL). Treatment with liraglutide was initiated, in addition to insulin therapy, at 0.6 mg every day for a week, titrated to 1.2 mg for the next week, and then to 1.8 mg daily. An insulin dose reduction of 20 units was recommended at the time that treatment with liraglutide was initiated. She reported no side effects, such as nausea, vomiting, diarrhea, or hypoglycemia. After 3 months of therapy, a total daily insulin dose of 200 units, metformin 2000 mg/day, and liraglutide 1.8 mg daily resulted in a much improved glycemic control (A1c 6.2%). She lost 1.8 kg. Similarly, the results of genetic tests for mutations in *LMNA* gene and *PPAR-gamma* gene were negative.

## Conclusions

A remarkable feature of lipodystrophic syndromes is the clinical heterogeneity regarding the extent and location of adipose tissue loss. Nevertheless, with very few exceptions, the majority of lipodystrophy disorders are associated with impaired insulin sensitivity and diabetes [8], which are complications that are difficult to treat with classical therapeutic options. In the two cases described, diabetes was successfully treated with the addition of a GLP-1 analogue to the previous therapeutic regimen. GLP-1 receptors (GLP-1R) are expressed in the arcuate and paraventricular nucleus of the hypothalamus, and recent evidence indicated that activation of arcuate GLP-1R sensitizes the liver to insulin and reinforces insulin action to suppress endogenous glucose production [11]. Furthermore, GLP-1 analogues seem to amplify insulin signaling in adipocytes by upregulating insulin signaling molecules. Liraglutide has been shown to ameliorate insulin resistance through upregulation of glucose transporter-4 and to decrease oxidative stress and inflammation. It has also been shown to improve endothelial and cardiac function, which might result in increasing systemic insulin sensitivity [12]. Data from studies in diabetic rats showed that exenatide inhibits hepatic gluconeogenesis and increases glucose uptake in the peripheral skeletal muscle with improvement of hepatic and extrahepatic insulin resistance [13]. According to some studies, these drugs significantly influence fat distribution, and even a short course of treatment results in a significant decrease of subcutaneous and visceral fat deposits [10, 14]. The effect starts showing shortly after the beginning of treatment [9, 15]. The extent of weight reduction is not always as significant as that of the result described in the first case, but it has been shown that important health benefits can be achieved with modest weight loss [10]. For patients taking insulin, the addition of GLP-1 analogue may help offset weight gain, or even promote some weight loss and reduce insulin needs, with an improvement in glycemic control [16]. In fact, in our second case, there was a small reduction in weight, although the patient achieved target levels of A1c with lower insulin requirements. The potential predictors of different weight loss response with GLP-1 analogues treatment remain unidentified. A study evaluating the impact of genetic variability of GLP-1R on weight response to liraglutide demonstrated that some polymorphisms are associated with individual differences regarding weight loss response in obese women with PCOS. Thus, as GLP-1R is a major target for the GLP-1 analogues, its genetic variability could be hypothetically linked with the different response to the weight-reducing potential of these drugs in a clinically homogenous population [17].

As far as we know, our cases are the first reports of the treatment of patients with lipodystrophic syndromes not human immunodeficiency vírus (HIV)-associated, with severe insulin resistance, with GLP-1 analogues. We intend to demonstrate that GLP-1 analogues could be used as new tools for the treatment of these metabolic and adipose tissue disorders, reducing fat mass and insulin resistance, decreasing insulin requirements, and improving A1c.

## Abbreviations

A1c: Glycated hemoglobin
BMI: Body mass index
GLP-1: Glucagon-like peptide-1
GLP-1R: GLP-1 receptors
HDL: High-density lipoprotein
HIV: Human Immunodeficiency Vírus
LDL: Low-density lipoprotein
PCOS: Polycystic ovary syndrome
